# Human Infection by Zoonotic Eye Fluke *Philophthalmus lacrymosus*, South America

**DOI:** 10.3201/eid3112.251126

**Published:** 2025-12

**Authors:** Thomas Weitzel, Esteban M. Cordero, Trinidad Mujica, Carolina Aravena, Brianne E. Phillips, Michael J. Yabsley, Gregory A. Lewbart, Diego Páez-Rosas, María Isabel Jercic, Sofía Capasso

**Affiliations:** Clínica Alemana, Universidad del Desarrollo, Santiago, Chile (T. Weitzel, C. Aravena); Charité–Universitätsmedizin Berlin, Berlin, Germany (T. Weitzel); Instituto de Salud Pública, Santiago (E.M. Cordero, M.I. Jercic), Fundación Instituto Professional Duoc UC, Santiago (T. Mujica); Zoo New England, Boston, Massachusetts, USA (B.E. Phillips); University of Georgia, Athens, Georgia, USA (M.J. Yabsley); North Carolina State University, Raleigh, North Carolina, USA (G.A. Lewbart), Universidad San Francisco de Quito, Puerto Baquerizo Moreno, Ecuador (D. Páez-Rosas); Oniris, INRAE, BIOPAR, Nantes, France (S. Capasso); Galápagos Conservancy, Puerto Ayora, Ecuador (D. Páez-Rosas)

**Keywords:** parasites, zoonoses, parasitic infection, trematode, trematodiasis, avian parasite, epidemiology, South America, vector-borne infections, Ecuador

## Abstract

We report a case of severe conjunctivitis in a traveler infected with a *Philophthalmus lacrymosus* eye fluke, probably acquired on the Galápagos Islands in Ecuador. This zoonotic parasite is endemic in Brazil and Venezuela, where it has been reported in birds and capybaras.

*Philophthalmus* spp. parasites are cosmopolitan digeneans, typically inhabiting the conjunctival sac of waterbirds. The trematodes, known as avian eye flukes, have a complex lifecycle, which includes freshwater and marine snails as intermediate hosts and waterbirds as final hosts. Birds are infected by ingestion of infective metacercariae that are encysted on aquatic plants (*1*). After thermally triggered excystation in the pharynx, the parasite migrates through the lacrimal ducts to the orbital cavity. Ocular infections of mammals have been reported in capybaras (*Hydrochoerus hydrochaeris*) from Brazil, Galapagos sea lions (*Zalophus wollebaeki*) in Ecuador, and rarely other mammals, including humans (*2*–*6*). The mode of infection in nonavian hosts is uncertain; both ingestion and direct contact with cercariae or metacercariae have been proposed (*2*,*7*).

Globally, >50 nominal *Philophthalmus* spp. trematodes have been described; however, recent evidence suggests that only ≈10 species are valid (*1*,*5*). In South America, *Philophthalmus* spp. flukes have been reported from Brazil, Peru, Venezuela, and Ecuador’s Galápagos Islands (*1*,*3*,*8*,*9*). Further geographic spread of *Philophthalmus* spp. trematodes by invasive snail species is probable (*9*,*10*).

Since 1939, a total of 12 human philophthalmiasis cases have been published. Infections were acquired in Asia, Europe, and North America and mostly identified to the genus level, but cases compatible with philophthalmiasis already were known in the 19th Century (Appendix 1). We describe a case of philophthalmiasis caused by a *Philophthalmus lacrymosus* eye fluke in a traveler from Europe, probably acquired on the Galápagos Islands.

## The Study

A 26-year-old woman from England sought care in Santiago, Chile, for a 9-day history of intense pain, swelling, and a moving foreign body sensation in her right eye. Before symptom onset, she had visited Colombia (4-week stay); Ecuador, including Galápagos Islands (2.5-week stay); and Peru (1-week stay). Ocular examination showed eyelid edema, intense chemosis and follicular reaction of the inferior fornix, and superior tarsal conjunctiva. Results of cornea examination, anterior segment findings, and fundus examination were unremarkable. After a thorough examination, we removed an elongated mobile structure located on the upper tarsal conjunctiva by using a moist cotton swab. After removal, the foreign body sensation disappeared. Follow-up over the following weeks showed a complete recovery without complications.

**Figure 1:**
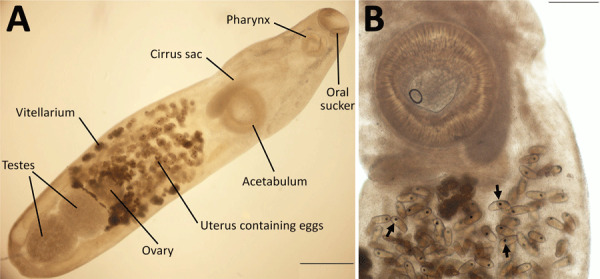
Specimen of *Philophthalmus lacrymosus* fluke extracted from conjunctiva of a female traveler from England in Chile. A) Full view of the unstained specimen showing oral sucker, pharynx, cirrus sac, acetabulum, uterus containing eggs, ovary, testes, and vitellarium. B) Intrauterine eggs showing fully formed miracidia with eyespots (arrows). Scale bars: panel A, 500 µm; panel B, 200 µm.

We performed detailed morphologic studies of the extracted structure on a temporary wet mount by using an Olympus SZ61 stereo microscope (https://www.olympus-global.com), an Olympus DP22 digital camera, and Olympus cellSens software version 2.3 (https://evidentscientific.com). Our analyses confirmed the specimen as a *P. lacrymosus* fluke (Figure 1). The single mature ovigerous specimen had an elongated oval shape, smooth surface, no spines, and constriction at the level of the ventral sucker. Maximum width was always posterior to ventral sucker, oral sucker subterminal, pharynx muscular, esophagus bifurcating posteriorly to pharynx, and ceca extending to posterior margin of posterior testis. The acetabulum was larger than the oral sucker and preequatorial; testes were in tandem, smooth and spherical, located in the posterior portion of the body, in an intercecal position; cirrus sac was elongated, slightly surpassing posterior margin of acetabulum (cirrus not visible), and genital pore median, at level of cecal bifurcation. Ovary was spherical, located pretesticular in the intercecal area; the uterus was long and coiled, occupying the space between the ventral sucker and the level of anterior margin of the anterior testis; the vitellarium was follicular and eggs nonoperculated, containing a fully formed miracidium with a dark eyespot in most eggs. We morphometrically compared the sample with previously reported *P. lacrymosus* specimens (Appendix 1).

We confirmed species diagnosis by molecular analyses using PCR and bidirectional Sanger sequencing of nuclear internal transcribed spacer (ITS) 2 and mitochondrial cytochrome c oxidase I (Cox1) (Appendix 2). We compared amplicons with sequences from GenBank and *Philophthalmus zalophi* ocular trematodes from Galápagos sea lions.

**Figure 2:**
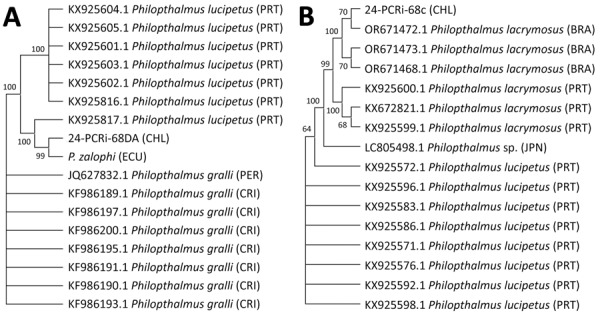
Maximum-likelihood phylogenetic tree constructed from 17 internal transcribed spacer 2 sequences (trimmed alignment 673 bp) (A) and 16 cytochrome c oxidase I sequences (trimmed alignment 365 bp) (B) of *Philophthalmus lacrymosus* fluke extracted from conjunctiva of a female traveler from England in Chile. Consensus trees were inferred from 1,000 replicates by using the Kimura 2-parameter test in MEGA 11 (https://www.megasoftware.net). Bootstrap values at the nodes indicate the percentages of replicates in which the sequences clustered together. Sequence codes include GenBank accession numbers and parasite information. In parentheses, letter codes indicate the country of origin. Sequences from this case report are 24-PCRi-68DA_CHL (GenBank accession no. PX240011) and 24-PCRi-68c_CHL (accession no. PX238763). BRA, Brazil; CHL, Chile; CRI, Costa Rica; ECU, Ecuador; JPN, Japan; PER, Peru; PRT, Portugal.

An 861-bp ITS-2 consensus sequence from the human sample was identical to the consensus *P. zalophi* sequence (M.J. Yabsley, unpub. data) and showed 98.6% identity with *P. lacrymosus* and 95.9% identity with *P. lucipetus*, both fluke species found in gulls (*Larus* spp.) from Portugal, and 95.6% identity with *P. gralli* sequences from invasive red-rimmed melania snails (*Melanoides tuberculata*) from Costa Rica and small passerines from Peru (Figure 2). The 396-bp Cox1 consensus sequence had 99.73% (single transition) and 99.45% identity with *P. lacrymosus* sequences from kelp gulls (*Larus dominicanus*) in Brazil, whereas similarities with *P. lacrymosus* sequences from Portugal were 91.90%–92.15%. We observed identity values ranging from 87.34% to 87.09% with *P. lucipetus* sequences from Portugal. An 87.12% identity was shared with a specimen annotated as *Philopththalmus* sp., which was isolated from a Japanese snail (*Semisulcospira libertina*) (Figure 2). We constructed additional Bayesian-inferred phylogenetic trees from 17 ITS-2 sequences and 16 Cox1 sequences (Appendix 1 Figure).

## Conclusions

The epidemiology of human philophthalmiasis is poorly understood. Cases have been reported from Asia, Europe, and North America (Appendix 1 Table 1). Some reports suggest infection by direct inoculation of metacercariae during swimming (Appendix 1), whereas others suggest oral ingestion of metacercaria with food or direct inoculation during food preparation (Appendix 1). Of note, the parasite can survive for several months in the human host (Appendix 1). Nearly all cases were caused by single worms and involved unilateral irritation, sensation of a foreign body, and conjunctivitis. Vision impairment has not been reported, except for historical cases in the 18th Century with high worm loads (Appendix 1).

The number of species causing human philophthalmiasis is uncertain, given that most extracted worms were identified only to genus. In North America, the *P. lacrymosus* fluke (recorded as *P. lacrimosus*) was diagnosed in a human case from Mexico (*11*). In South America, *P. lacrymosus* flukes have exclusively been diagnosed in waterbirds in Brazil and Venezuela and capybaras in Brazil (*5*). The patient reported here had only direct contact with natural water environments on the Galápagos Islands, where *P. zalophi*, a new fluke species defined by morphologic criteria, has been reported in Galapagos sea lions (*3*,*4*). Considering that *Philophthalmus* parasites can inhabit the human eye for several months, a previous case from Ohio, USA, might plausibly have also been acquired on the Galápagos Islands, which the patient had visited 5 months before symptom onset (*12*). Marine snails of the Batillariidae family, present on the Galápagos Islands, could serve as potential intermediate hosts; that family includes the West Indian false cerith (*Lampanella minima*), a known intermediate host of *P. lacrymosus* flukes (*13*,*14*).

The patient’s exposure on Galápagos Islands remains inferential, requiring studies of infected intermediate hosts or environmental larval stages. However, our molecular data indicate that *P. zalophi* flukes from Galápagos might be conspecific with *P. lacrymosus* flukes. Probable spillover of *P. lacrymosus* flukes from a bird host to sea lions could explain morphologic differences, which can occur during adaptation to the mammalian host as reported in capybaras (*2*). Similarly, host-related plasticity or different development stages could explain certain morphologic deviations of our sample from previous *P. lacrymosus* specimens (Appendix 1). The specimen we report shared several traits with *P. zalophi* flukes, including a similar oral sucker to pharynx ratio and a body length <6 mm.

Further comparative genomic analyses are required to clarify taxonomic uncertainty of *Philophthalmus* spp. flukes infecting humans, as recently shown in Japan (*15*). The *P. lacrymosus* species might be paraphylectic or represent a complex of geographically and host-related lineages with South American isolates forming a genetically cohesive clade that is taxonomically distinct from forms of *P. lacrymosus* flukes in Europe and Asia.

In conclusion, our clinical and epidemiologic findings show that the zoonotic eye fluke *P. lacrymosus* can infect humans in South America. The findings also suggest that the parasite might be endemic on the Galápagos Islands in Ecuador.

Appendix 1 Additional methods for human infection by zoonotic eye fluke *Philophthalmus lacrymosus*, South America.

Appendix 2 Additional information about human infection by zoonotic eye fluke *Philophthalmus lacrymosus*, South America.
