# Supplementary material for: Human Infection by Zoonotic Eye Fluke Philophthalmus lacrymosus, South America
Source: Emerg Infect Dis. 2025 Dec;31(12):2293–6. doi: 10.3201/eid3112.251126 (PMC12782273; doi:10.3201/eid3112.251126)
Supplement: Appendix 1 — Additional methods for human infection by zoonotic eye fluke Philophthalmus lacrymosus, South America. [file 25-1126-Techapp-s1.pdf]

Article DOI: https://doi.org/10.3201/eid3112.251126

EID cannot ensure accessibility for supplementary materials supplied by authors. Readers who have difficulty accessing supplementary content should contact the authors for assistance.

Human Infection by Zoonotic Eye Fluke *Philophthalmus lacrymosus*, South America

Appendix 1

Human Philophthalmiasis Cases

Information on previously published human philophthalmiasis cases is summarized (Appendix 1 Table 1). Some reports include detailed information on the patients' exposure history suggesting that infection can occur through direct inoculation of metacercariae during water contact (swimming) (*6*) or through oral ingestion of metacercariae (*8,11*). *Philophthalmus* flukes might survive for several months in the human host (*3,6,9*). One severe case with high worm loads caused visual impairment (*13*).

Additional Molecular Methods and Results

Molecular Analyses of Human Sample (Chile)

To maintain the integrity of the specimen, total DNA was purified directly from the transport medium (70% ethanol). A 500 µL aliquot was transferred to a clean tube and centrifuged to 14,000 rpm for 10 minutes to sediment any cellular debris. The supernatant was discarded, and the pellet was air-dried. The dried material was then resuspended in 200 µL of ATL buffer, and the DNA extracted using the QIAamp Mini Kit (Cat. 51306) following the manufacturer's instructions. The purified nucleic acids were eluted in 100 µL of AE buffer.

Molecular identification was performed via PCR amplification of the nuclear ITS-2 and mitochondrial Cox1 gene regions, using primers and cycling conditions previously described (*15*,*16*). Amplicons were resolved on agarose gels, purified and subjected to bidirectional Sanger sequencing. Assembled contigs were matched against the GenBank nucleotide database using the BLASTn tool (*17*). The top fifteen sequences with the highest similarity scores were retrieved,

trimmed to the maximum common length (i.e., 673 bp for ITS-2 and 365 bp for Cox1) and used to construct maximum likelihood phylogenetic trees in MEGA 11 software using Kimura 2-parameter model and 1,000 bootstrap replicates.

Molecular Analyses of Samples from Sea Lions

Three specimens of *Philophthalmus zalophi* previously collected from Galápagos sea lions (*Zalophus wollebaeki*) and preserved in 70% ethanol were processed for molecular analysis. Prior to DNA extraction, the flukes were dried in a biosafety cabinet overnight to remove residual ethanol. Genomic DNA was extracted using the DNeasy Blood and Tissue Kit (Qiagen, Hilden, Germany), following the manufacturer's protocol for tissue. The 18S rDNA-ITS1–5.8S rRDA-ITS-2 region was amplified with primers NSF1419/20, Dig.5.8SR, NC13(ITS2)/F, and Dd28SR1, as previously described (*14*).

Amplicons were visualized in 2% agarose gels stained with GelRed (Biotium, Hayward, California), then excised and purified using the QIAquick gel extraction kit (Qiagen). Purified amplicons were submitted to GeneWiz Corporation (South Plainfield, New Jersey, USA) for bi-directional Sanger sequencing. Chromatographs were analyzed using Geneious Prime v2021.1.1 (Auckland, New Zealand, https://www.geneious.com).

All three *P. zalophi* individuals had identical ITS-2 sequences.

Additional Molecular Results of Chilean Sample

Additional analyses on the ITS-2 and Cox1 sequences retrieved from the Chilean sample and from GenBank are provided (Appendix 1 Tables 2, 3) (Appendix 2 Tables 1, 2, https://wwwnc.cdc.gov/EID/article/31/12/25-1126-App2.xlsx). Full-length consensus contigs for newly sequenced IT2–2 and Cox1 nt data obtained in this work were submitted to GenBank under accession numbers PX240011 and PX238763, respectively. Additional Bayesian-inferred phylogenetic analyses are shown (Appendix 1 Figure).

Estimates of evolutionary divergence between ITS2 and COX1 sequences are shown (Appendix 2 Tables 1, 2).

Morphological Details

Morphological details of the Chilean human specimen and comparison with previous reports of *P. lacrymosus* are shown (Appendix 1 Table 4). Its body length was shorter compared

to samples from Brazil and Mexico, and the acetabulum, pharynx, and cirrus sac were smaller than in specimens from gulls or sea lions. These differences may reflect different developmental stages or host-related influences. Such host-induced morphological plasticity in *P. lacrymosus* has been documented (*23*,*24*). Conversely, features such as the genital pore located posterior to the intestinal bifurcation, tandem testes, a pretesticular ovary, and consistent OS/A and OS/PH ratios were shared across specimens from different hosts (*7*,*18*,*23*,*25*).

References

1. Marcovic A. Der erste Fall von Philophthalmose beim Menschen. Von Graefes Arch Ophthal. 1939;140:515–20.
2. Dissanaike AS, Bilimoria DP. On an infection of a human eye with *Philophthalmus* sp. in Ceylon. J Helminthol. 1958;32:115–8. PubMed
3. Kalthoff H, Janitschke K, Mravak S, Schopp W, Werner H. Mature avian eye fluke (*Philophthalmus* sp.) under the human conjunctiva [in German]. Klin Monbl Augenheilkd. 1981;179:373–5. **PMID 7339163**
4. Mimori T, Hirai H, Kifune T, Inada K. *Philophthalmus* sp. (Trematoda) in a human eye. Am J Trop Med Hyg. 1982;31:859–61. **PMID 7102921**
5. Gutierrez Y, Grossniklaus HE, Annable WL. Human conjunctivitis caused by the bird parasite *Philophthalmus.* Am J Ophthalmol. 1987;104:417–9. **PMID 3661653**
6. Lang Y, Weiss Y, Garzozi H, Gold D, Lengy J. A first instance of human philophthalmosis in Israel. J Helminthol. 1993;67:107–11. PubMed
7. Lamothe-Argumedo R, Diaz-Camacho SP, Nawa Y. The first human case in Mexico of conjunctivitis caused by the avian parasite, *Philophthalmus lacrimosus.* J Parasitol. 2003;89:183–5. **PMID 12659326**
8. Waikagul J, Dekumyoy P, Yoonuan T, Praevanit R. Conjunctiva philophthalmosis: a case report in Thailand. Am J Trop Med Hyg. 2006;74:848–9. **PMID 16687691**
9. Basak SK, Singhal P, Hazra TK, Gibson DI. Avian trematode *Philophthalmus.* Ophthalmology. 2006;113:1063.e1–2. **PMID 16751053**
10. Rajapakse RD, Wijerathne KMS. S de Wijesundera M. Ocular infection with an avian trematode (*Philophthalmus* sp). Ceylon Med J. 2009;54:128–9. **PMID 20052855**

11. Sato C, Sasaki M, Nabeta H, Tomioka M, Uga S, Nakao M. A philophthalmid eyefluke from a human in Japan. J Parasitol. 2019;105:619–23. **PMID 31418651**

12. Sapp SGH, Alhabshan RN, Bishop HS, Fox M, Ndubuisi M, Snider CE, et al. Ocular trematodiasis caused by the avian eye fluke *Philophthalmus* in southern Texas. Open Forum Infect Dis. 2019;6:ofz265. PubMed

13. Kanev I, Nollen PM, Vassilev I, Radev V, Dimitrov V. Redescription of *Philophthalmus lucipetus* (Rudolphi. (Trematoda: Philophthalmidae) with a discussion of its identity and characteristics. Ann Naturhist Mus Wien. 1819;94(95B):11–34.

14. Sasaki M, Miura O, Nakao M. *Philophthalmus hechingeri* n. sp. (Digena: Philophthamidae), a human-infecting eye fluke from the Asian mud snail, *Batillaria attramentaria.* J Parasitol. 2022;108:44–52. **PMID 35038324**

15. Otranto D, Rehbein S, Weigl S, Cantacessi C, Parisi A, Lia RP, et al. Morphological and molecular differentiation between *Dicrocoelium dendriticum* (Rudolphi, 1819) and *Dicrocoelium chinensis* (Sudarikov and Ryjikov, 1951) Tang and Tang, 1978 (Platyhelminthes: Digenea). Acta Trop. 2007;104:91–8. **PMID 17803950**

16. Bowles J, Blair D, McManus DP. Genetic variants within the genus *Echinococcus* identified by mitochondrial DNA sequencing. Mol Biochem Parasitol. 1992;54:165–73. **PMID 1435857**

17. Altschul SF, Gish W, Miller W, Myers EW, Lipman DJ. Basic local alignment search tool. J Mol Biol. 1990;215:403–10. **PMID 2231712**

18. Heneberg P, Casero M, Waap H, Sitko J, Azevedo F, Těšínský M, et al. An outbreak of philophthalmosis in *Larus michahellis* and *Larus fuscus* gulls in Iberian Peninsula. Parasitol Int. 2018;67:253–61. **PMID 29288885**

19. Heneberg P, Rojas A, Bizos J, Kocková L, Malá M, Rojas D. Focal *Philophthalmus gralli* infection possibly persists in *Melanoides tuberculata* over two years following the definitive hosts' removal. Parasitol Int. 2014;63:802–7. PubMed

20. Literák I, Heneberg P, Sitko J, Wetzel EJ, Cardenas Callirgos JM, Čapek M, et al. Eye trematode infection in small passerines in Peru caused by *Philophthalmus lucipetus*, an agent with a zoonotic potential spread by an invasive freshwater snail. Parasitol Int. 2013;62:390–6. **PMID 23570701**

21. Hernández DL, Somma AT, Steuernagel A, Vieira TSWJ, Moore B, Reifur L, et al. A molecular phylogenetic study of the eye fluke *Philophthalmus lacrymosus* (Trematoda: Philophthalmidae)

found in *Larus dominicanus* (Aves: Laridae) from Brazil. Acta Parasitol. 2024;69:1027–34. **PMID 37989828**

22. Miura O, Takisawa S. Biogeography of larval trematodes in the freshwater snail, *Semisulcospira libertina*: a comparison of the morphological and molecular approaches. Parasitol Int. 2024;102:102924. PubMed

23. Somma AT, Steuernagel A, Pulido-Murillo EA, Pinto HA, Reifur L, Moore BA, et al. Ocular disease caused by the trematode *Philophthalmus lachrymosus* in free-living kelp gulls (*Larus dominicanus*) of Brazil. J Vet Med Sci. 2022;84:1447–52. **PMID 36047247**

24. Pinto RM, dos Santos LC, Tortelly R, Menezes RC, de Moraes W, Juvenal JC, et al. Pathology and first report of natural infections of the eye trematode *Philophthalmus lachrymosus* Braun, 1902 (Digenea, Philophthalmidae) in a non-human mammalian host. Mem Inst Oswaldo Cruz. 2005;100:579–83. PubMed

25. Nasir P, Díaz MT, Lemus de Guevara D. Avian flukes of Venezuela. Riv Parassitol. 1972;33:245–76.**PMID 5529541**

Appendix 1 Table 1. Published cases of human philophthalmiasis

Year	Country	Gender	Age (yrs)	Clinical presentation	Diagnosis	Methods	Ref.
1939	Serbia	Male	?	Follicular conjunctivitis	*P. lucipetus**	Morphology	(*1*)
1956	Sri Lanka	Male	52	Conjunctivitis	*Philophthalmus* sp.	Morphology	(*2*)
1980	Sri Lanka	Male	27	Brown conjunctival lesion	*Philophthalmus* sp.	Morphology	(*3*)
1981	Japan	Male	67	Conjunctivitis	*Philophthalmus* sp.†	Morphology	(*4*)
1987	USA	Male	66	Follicular conjunctivitis‡	*Philophthalmus* sp.	Morphology	(*5*)
1991	Israel	Female	13	Severe conjunctivitis	*Philophthalmus* sp.	Morphology	(*6*)
2002	Mexico	Male	31	Conjunctivitis	*P. lacrymosus*	Morphology	(*7*)
2005	Thailand	Female	31	Conjunctivitis	*Philophthalmus* sp.	Morphology	(*8*)
2006	India	Male	18	Conjunctivitis	*Philophthalmus* sp.	Morphology	(*9*)
2009	Sri Lanka	Female	32	Conjunctivitis, episcleritis	*Philophthalmus* sp.	Morphology	(*10*)
2017	Japan	Female	64	Acute conjunctivitis left eye	*Philophthalmus* sp.†	Morphology/Molecular	(*11*)
2019	USA	Male	47	Subconjunctival hemorrhage	*Philophthalmus* sp.	Morphology	(*12*)
2024	South America§	Female	26	Severe conjunctivitis	*P. lacrymosus*	Morphology/Molecular	Chile¶

*Initially reported as *P. lacrymosus* but redescribed as *P. lucipetus* by Kanev et al., these authors mention 18 possible human cases during 1832–1919 in Europe (*13*).
†A newly described species was proposed to be responsible for the human cases in Japan: *Philophthalmus hechingeri* n. sp (*14*).
‡Patient traveled to Galápagos Islands 5 mo before symptom onset.
§Exposure in Colombia or Ecuador, most probably on Galápagos Islands.
¶Patient of this study.

Appendix 1 Table 2. BLASTn top fifteen sequences with the highest similarity to ITS-2 contig.

GenBank accession	Annotation	Identity (%)	Host	Country	Ref.
KX672817.1	*P. lacrymosus*	98.60	*Larus fuscus*	Portugal	(*18*)
KX925602.1	*P. lucipetus*	95.93	*Larus michahellis*	Portugal	(*18*)
KX925604.1	*P. lucipetus*	95.93	*Larus fuscus*	Portugal	(*18*)
KX925601.1	*P. lucipetus*	95.93	*Larus fuscus*	Portugal	(*18*)
KX672816.1	*P. lucipetus*	95.93	*Larus fuscus*	Portugal	(*18*)
KX925605.1	*P. lucipetus*	95.93	*Larus michahellis*	Portugal	(*18*)
KX925603.1	*P. lucipetus*	95.93	*Larus michahellis*	Portugal	(*18*)
KF986197.1	*P. gralli*	95.69	*Melanoides tuberculata*	Costa Rica	(*19*)
KF986195.1	*P. gralli*	95.69	*Melanoides tuberculata*	Costa Rica	(*19*)
KF986190.1	*P. gralli*	95.69	*Melanoides tuberculata*	Costa Rica	(*19*)
KF986200.1	*P. gralli*	95.69	*Melanoides tuberculata*	Costa Rica	(*19*)
KF986193.1	*P. gralli*	95.69	*Melanoides tuberculata*	Costa Rica	(*19*)
KF986191.1	*P. gralli*	95.69	*Melanoides tuberculata*	Costa Rica	(*19*)
JQ627832.1	*P. gralli*	95.69	*Tachuris rubrigastra*	Peru	(*20*)
KF986189.1	*P. gralli*	95.69	*Melanoides tuberculata*	Costa Rica	(*19*)

Appendix 1 Table 3. BLASTn top fifteen sequences with the highest similarity to COX I contig.

GenBank accession	Annotation	Identity (%)	Host	Country	Ref.
OR671472.1	*P. lacrymosus*	99.73	*Larus domicanus*	Brazil	(*21*)
OR671473.1	*P. lacrymosus*	99.45	*Larus domicanus*	Brazil	(*21*)
OR671468.1*	*P. lacrymosus*	99.45	*Larus domicanus*	Brazil	(*21*)
KX672821.1	*P. lacrymosus*	92.15	*Larus fuscus*	Portugal	(*18*)
KX925599.1	*P. lacrymosus*	92.15	*Larus fuscus*	Portugal	(*18*)
KX925600.1	*P. lacrymosus*	91.90	*Larus fuscus*	Portugal	(*18*)
KX925572.1	*P. lucipetus*	87.34	*Larus fuscus*	Portugal	(*18*)
LC805498.1	*Philophthalmus sp.*	87.12	*Semisulcospira libertina*	Japan	(*22*)
KX925583.1	*P. lucipetus*	87.09	*Larus fuscus*	Portugal	(*18*)
KX925596.1	*P. lucipetus*	87.09	*Larus fuscus*	Portugal	(*18*)
KX925586.1	*P. lucipetus*	87.09	*Larus michahellis*	Portugal	(*18*)
KX925592.1	*P. lucipetus*	87.09	*Larus fuscus*	Portugal	(*18*)
KX925571.1	*P. lucipetus*	87.09	*Larus fuscus*	Portugal	(*18*)
KX925576.1	*P. lucipetus*	87.09	*Larus michahellis*	Portugal	(*18*)
KX925598.1	*P. lucipetus*	87.09	*Larus michahellis*	Portugal	(*18*)

*3 additional identical sequences, i.e., OR671469.1, OR671470.1 and OR671471.1

Appendix 1 Table 4. Morphological details of *Philophthalmus lacrymosus* specimens from the present case and previous studies

Definitive host	Human	Human	Kelp gull (*Larus dominicanus)*	Lesser black-backed gull (*Larus fuscus)*	Willet (*Tringa semipalmata)*	Capybara (*Hydrochaeris hydrochaeris)*
Reference	Present study	(*7*)	(*18*)	(*21*)	(*20*)	(*19*)
Locality	Chile	Mexico	Brazil	Portugal	Venezuela	Brazil
Body length	3985	5860	4282	2095	4000	3730
Body width	783	1530	1549	633	1500	1080
Oral sucker length	305	480	407	241	416	300
Oral sucker width	240	400	310	210	416	260
Acetabulum length	505	720	694	385	782	700
Acetabulum width	513	690	664	373	782	670
Pharynx length	256	498	415	241	543	200
Pharynx width	229	550	306	186	543	110
Esophagus length	239	-	-	86	-	-
Testicle ant length	553	210	537	270	347	370
Testicle ant width	446	190	417	254	347	230
Testicle post length	457	250	515	283	347	360
Testicle post width	448	240	451	271	347	280
Cirrus sac length	913	-	948	-	-	1670
Cirrus sac width	158	-	229	-	-	-
Ovary length	250	200	254	227	400	210
Ovary width	262	160	203	155	400	190
Eggs length	83	80	75	72	-	100
Eggs width	34	30	34	34	-	30
Vitellarium	follicular	follicular	follicular	follicular	follicular	follicular
Vitellarium follicular	5 (right), 4 (left)	4–5 follicles	-	-	-	-
OS/A	1:1.6	1: 1.8	1:2.2	1:1.8	1:1.8	1:3.3
OS/PH	1:1.2	1: 1.4	1:1.3	1:1.1	1:1.3	1: 0.7

Measurements in μm. AC, Acetabulum, OS, oral sucker, PH, pharynx.

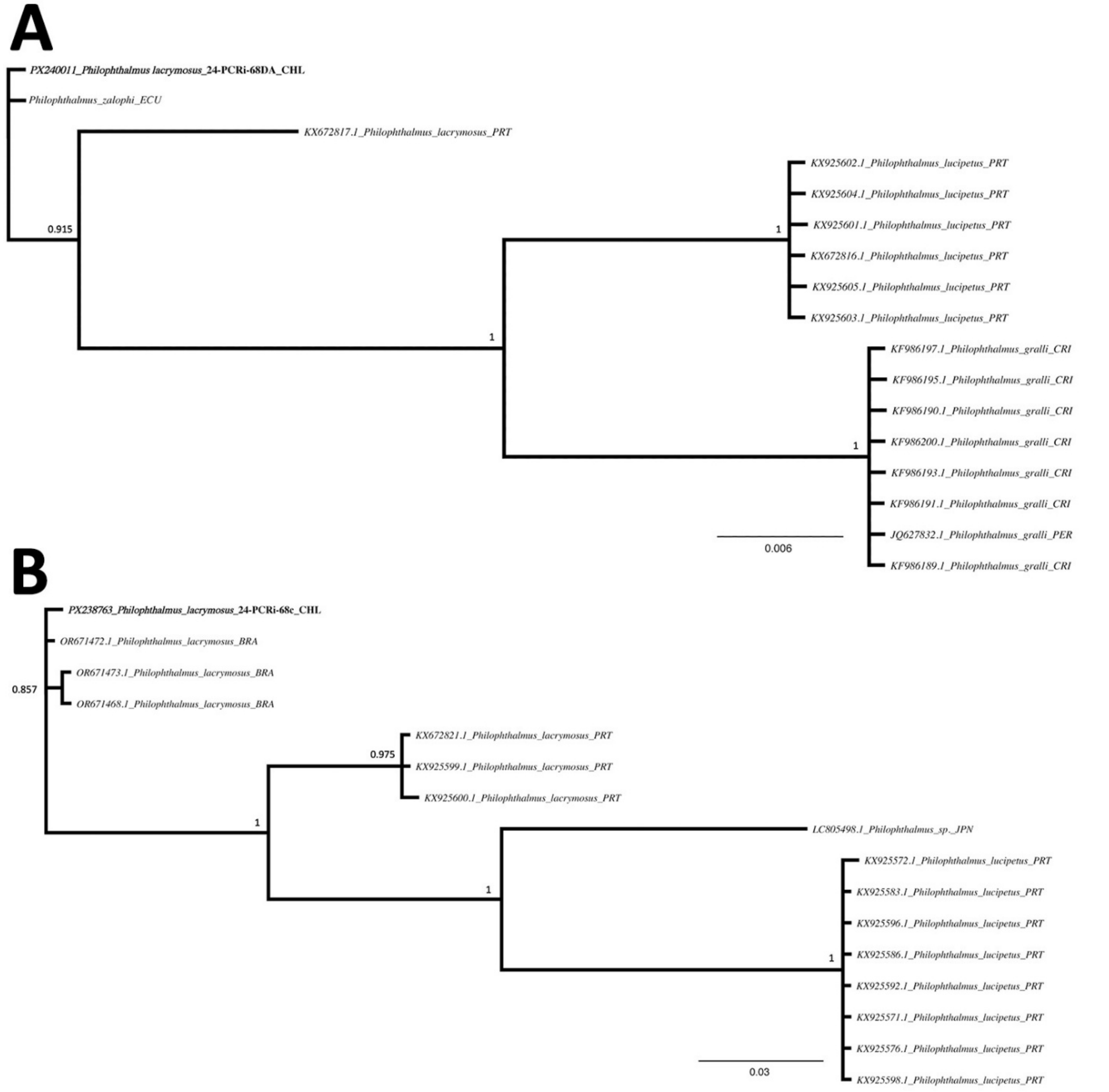

Appendix 1 Figure. Bayesian-inferred phylogenetic trees constructed from 17 ITS-2 sequences (A) and 16 Cox1 sequences (B). Phylogenetic trees were inferred using MrBayes v3.2.6 with the 4by4 substitution model. Markov Chain Monte Carlo (MCMC) algorithm was run for 100,000 generations sampling every 100 generations. The first 250 trees were discarded as burn-in before summarizing the results. The numbers in the nodes indicate the posterior probabilities. Three letter codes indicate the country of each isolate: CHL, Chile, ECU, Ecuador, PRT, Portugal, CRI, Costa Rica, PER, Peru, BRA, Brazil, and JPN, Japan. Lower bar on the left indicates the expected changes per site. PX240011_ *Philophthalmus lacrymosus*_24-PCRi-68DA_CHL and PX238763_ *Philophthalmus lacrymosus* 24-PCRi-68c_CHL, specimen from this case report. Lower bar indicates the expected changes per site.
